# Inconsistency in cervical dislocation: A UK survey of techniques and tools utilised for laboratory rodents

**DOI:** 10.1017/awf.2026.10087

**Published:** 2026-04-29

**Authors:** Jessica E. Martin, Matthew C. Leach, Jasmine M. Clarkson

**Affiliations:** 1 School of Natural and Environmental Sciences, Newcastle University, United Kingdom; 2 Comparative Biology Centre, Newcastle University, United Kingdom

**Keywords:** Animal welfare, euthanasia, humane, killing, laboratory rodents, Schedule 1

## Abstract

Rodents remain the predominant mammalian species used for biomedical research and must be humanely killed upon completion of the scientific work. Across the UK, cervical dislocation is reported as the most common method for humanely killing laboratory rodents. Cervical dislocation involves the separation of the vertebrae at the top of the spine and can be achieved manually or mechanically (e.g. using a tool). There is no standardised method for achieving consistent cervical dislocation in the desired location, and no dedicated tool specifically designed, validated and commercially available to achieve accurate and effective dislocation. Previous work has highlighted inaccuracy in method application and, as such, has the potential to risk animal welfare at killing. The aim of this work was to identify the techniques used by personnel across UK institutions for performing cervical dislocation using an online questionnaire. We found marked inconsistencies in technique and the use of tools to aid the application. Mice are predominantly killed via manual operation (i.e. without the aid of a tool), while rats are more often killed using mechanical aids. A wide range of improvised tools (i.e. not designed for killing) were reported, including pens, scissors, and cage scrapers. Further, there was little or no consensus regarding which physical actions are essential for a successful dislocation (i.e. a stretch and/or a twist), and a lack of reported institutional standard operating procedures. Further work is needed to establish validated methods and clear standards to ensure this common method is applied humanely and consistently.

## Introduction

Rodents remain the predominant mammalian species used for biomedical research, accounting for approximately 70% of all experimental procedures across the UK in 2023 (Home Office 2025). An unfortunate consequence of their use in research is the requirement to be humanely killed after completion of the scientific study, if they are surplus or at the end of their breeding life. In the UK, killing laboratory rodents is regulated by Schedule 1 of the Animals (Scientific) Procedures Act (1986) (Home Office 1986). This schedule lists permitted methods according to the species, age and weight. For laboratory rodents these methods include overdose of an anaesthetic, exposure to carbon dioxide gas, dislocation of the neck (in rodents up to 500 g with prior sedative or anaesthetic in rodents over 150 g) and concussion of the brain by striking the cranium (Home Office 1986).

From these permitted methods, we recently highlighted (Clarkson *et al.* 2023) that across the UK, cervical dislocation is the most common killing method employed, with 57.5% of participants stating that cervical dislocation was their preferred method. Cervical dislocation involves the separation of the vertebrae at the top of the spine and can be achieved manually (e.g. using thumb and forefinger) or mechanically (i.e. using a dedicated tool or device). However, currently there is no standardised method for achieving consistent cervical dislocation in the desired location, and no dedicated tool specifically designed and commercially available to achieve accurate and effective dislocation. As such, research institutions commonly employ different manual or mechanical methods, including unvalidated tools, whose effectiveness and animal welfare implications remain unassessed. Therefore, the lack of consistency across and within institutions poses a major risk to animal welfare.

To ensure rapid loss of consciousness and death, cervical dislocation should occur as high as possible on the spinal column, ideally between the atlanto-occipital joint and the first cervical vertebrae (C1) (Carbone *et al.* 2012). The few studies that have been undertaken to assess location of the separation, have been predominantly conducted in mice (*Mus musculus*; Cartner *et al.* 2007; Carbone *et al.* 2012; Davidge *et al.* 2025). These studies highlighted the inherent inaccuracy of the method to target the high spinal region, with only 78% of mice having thoracic spinal dislocation in addition to the desired cervical location (Cartner *et al.* 2007). If this is indeed a true reflection of what is happening in practice across and within institutions, then this poses serious and obvious consequences for animal welfare. It is possible that inaccurate dislocations result in longer times to death and unconsciousness, and may be associated with unnecessary pain and suffering.

The aim of this work was to identify the techniques used by personnel working across UK institutions for performing cervical dislocation using a short online questionnaire. We aimed to identify the prevalence of manual versus mechanical cervical dislocation, and for those utilising mechanical methods, identify the tools commonly used for both rats and mice.

## Materials and methods

### Participants

The target population for this survey was individuals who work for a research institution in the UK and who predominantly work with laboratory rodents (mice and/or rats [*Rattus norvegicus*]). The survey was piloted internally with target respondents (e.g. technicians) to ensure clarity in question phrasing and interpretation prior to dissemination. Participants were invited to take part via the dissemination of a link across various animal care, welfare and 3Rs organisations (e.g. NC3Rs, IAT, LASA, LAVA). Participation was voluntary, with no incentive offered. All participants who completed the survey were included in the analysis, and the research was approved by the Newcastle University ethics committee (Ref: 23-026-MAR).

### Online survey

The online survey was hosted through Jisc online surveys (v2) and was open between 21/08/2023 and 20/12/2023 (for the full survey, see Supplementary material). The survey was fully anonymous, and informed consent was obtained prior to commencement to the online questions. The survey had nineteen questions in total. The first part of the survey obtained basic demographic information from participants across four questions including which sector they currently worked in, their current role, their number of years’ experience working with laboratory rodents and the species they predominantly work with. The second and third parts consisted of identifying the most common techniques, including tools utilised for the cervical dislocation of laboratory mice and rats, respectively. The second and third sections also asked in the individual’s opinion whether cervical dislocation relied on any of several listed criteria (e.g. pulling the tail horizontally) and asked the extent to which they agree or disagree that cervical dislocation is humane for both mice and rats. For full survey questions and pre-determined listed answers, please refer to the Supplementary material.

### Data analysis

Data were exported from Jisc in an Excel® file format (.csv) and were subsequently processed and analysed in R and R Studio (version 2023.03.1+446). Results were fully anonymous, with no identifiable information collected and therefore in accordance with General Data Protection regulation (GDPR 2018). Only responses from individuals completing 100% the survey were included in data processing and subsequent statistical analyses. All data were collated and processed in R using the tidyverse package (Wickham *et al.* 2019). All graphical summaries were created using ggplot2 (Wickham 2016). Ranked data (e.g. degree of humaneness) were analysed using Cumulative Link Models (CLMs) using the packages ordinal (Haubo & Christensen 2019) and RVAideMemoire (Herve 2025) to compare the mean ranks with the threshold set to equidistant. Method (2 levels: manual or mechanical) was included as a fixed effect to determine its influence on humaneness rankings. In addition, demographic factors including: job role (5 levels); job sector (4 levels); and experience (6 levels) were included as fixed factors to determine their influence on humaneness ranking. Binary data were analysed using generalised linear mixed models using the package lme4 (Bates *et al.* 2015) with family set to binomial. An exploration of the influence of demographic factors and cervical dislocation method (2 levels: manual or mechanical) were performed via models including fixed factors such as job role (5 levels), job sector (4 levels), experience (6 levels). Binomial generalised linear mixed models were used to determine whether demographic factors influenced the likelihood of using manual or mechanical methods, of using a given tool, or of selecting a given criterion for successful cervical dislocation. Model fit was evaluated using the AIC and the DHARMa (Diagnosis for Hierarchical Multi-Level/Mixed regression Models) package to create diagnostic QQ residual plots. Statistical significance was based on *P* < 0.05 threshold on the Χ2 statistical test. Pair-wise comparisons were reported using estimated marginal means via the emmeans package, with *P*-values adjusted for multiplicity using the Tukey method (Lenth 2021) where non-significant results are not reported.

## Results

### Participant demographics

Table 1 summarises the participant demographics according to their reported sector, role and level of experience. A total of 317 participants completed the survey in full, with the majority coming from the academic sector, and a smaller proportion of participants reported coming from contract research organisations, the pharmaceutical industry or other sectors. We also asked individuals their current role and overall found a relatively even split between researchers and technicians, with other participants holding a veterinary, regulatory or other role. We also found a wide range in experience level across participants, ranging from 0–1 years to 24+ years.

**Table 1.** Summary of participant demographics (n = 317 respondents) according to reported sector, role and experience

	Number of respondents	Percentage of respondents (%)
Sector		
Academic	264	83.28
Contract Research Organisation	30	9.46
Pharmaceutical	11	3.47
Other	9	2.84
Role		
Researcher	155	48.90
Technician	123	38.80
Veterinary	18	5.67
Regulatory	3	0.95
Other	18	5.68
Experience		
0–1 years	10	3.15
2–5 years	76	23.97
6–11 years	74	23.34
12–17 years	56	17.67
18–23 years	46	14.51
24+ years	55	17.35

**Figure 1.**
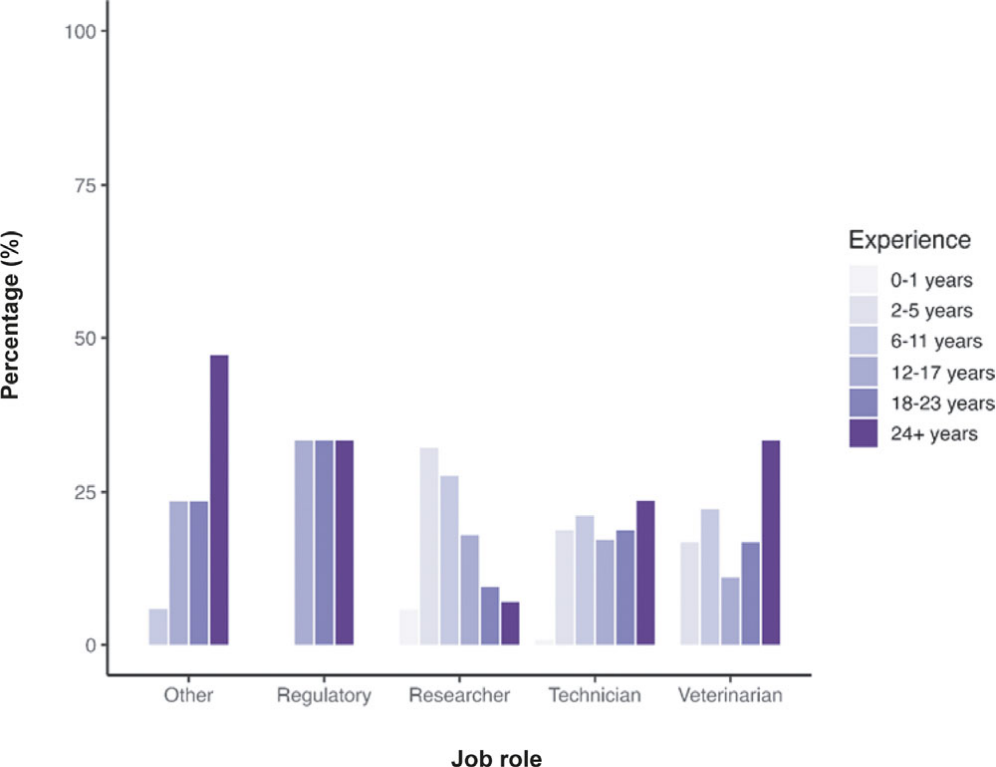
Percentage (%) of a total of 317 participant responses within each current job role according to their duration of experience working with laboratory rodents.

**Figure 2.**
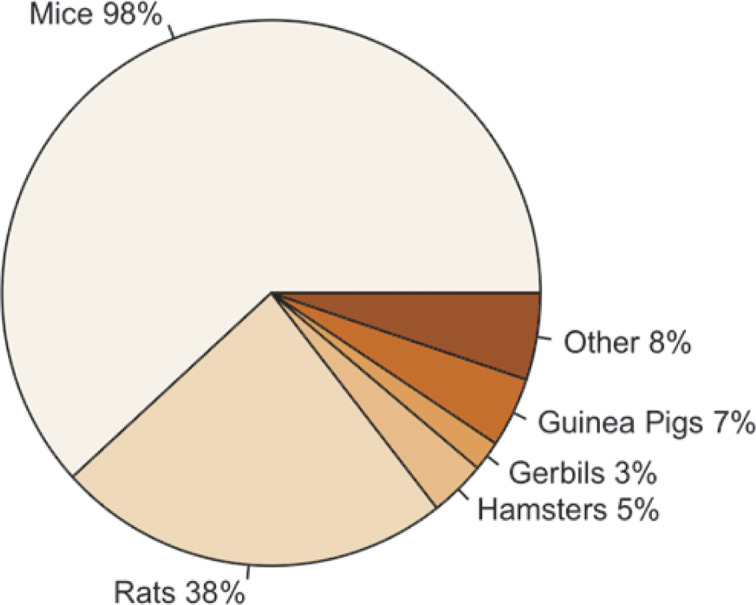
Percentage of participants (total n = 317) working with each of the following species; mice ([*Mus musculus*]; n = 312/317), rats ([*Rattus norvegicus*]; n = 120/317), hamsters ([*Mesocricetus auratus*]; n = 17/317), gerbils ([*Meriones unguiculatus*]; n = 9/317), guinea pigs ([*Cavia porcellus*]; n = 21/317) and other (n = 26/317).

Figure 1 highlights the spread of experience levels across each job role, demonstrating that technicians have a more balanced range of experience levels compared to researchers, who generally report lower experience levels. Individuals holding a regulatory role report experience levels > 6 years, and individuals in veterinary and other roles reported higher experience levels. In line with our previous survey (Clarkson *et al.* 2023),

Figure 2 highlights that the majority of participants primarily reported working with mice (*Mus musculus*); however, other rodent species were also reported, including rats (*Rattus norvegicus*), hamsters (*Mesocricetus auratus*), gerbils (*Meriones unguiculatus*), and guinea pigs (*Cavia porcellus*). Some participants also reported working with other species, including rabbits (*Oryctolagus cuniculus*), fish (e.g. Zebra fish [*Danio rerio*]), pigs (*Sus scrofa*), sheep (*Ovis aries*), dogs (*Canis lupus familiaris*) and non-human primates (e.g. Rhesus macaque [*Macaca mulatta*]), along with rodent species.

### Cervical dislocation techniques for laboratory mice

**Figure 3.**
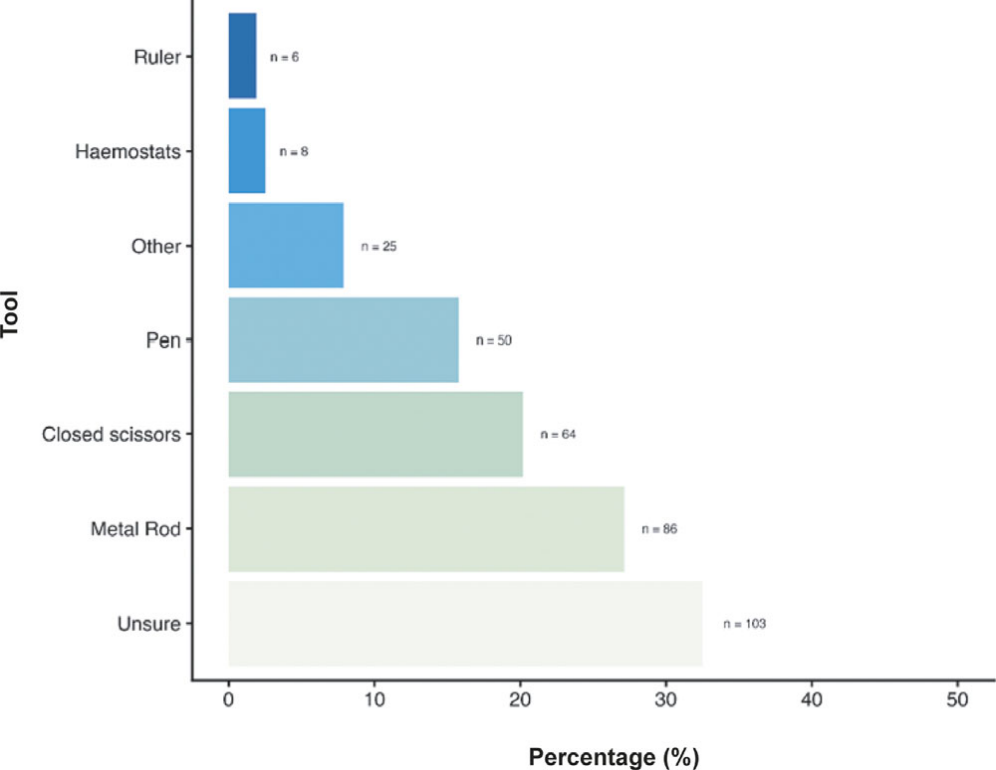
Percentage (%) of mechanical tools commonly used for cervical dislocation of those working with laboratory mice (*Mus musculus*; total; n = 312).

When asked about the primary technique used for cervical dislocation with laboratory mice, 69.4% (n = 220/312) reported using manual methods (e.g. thumb and forefinger with no dedicated equipment), 25.9% (n = 82/312) reporting mechanical methods (e.g. methods using dedicated tools) and 3.2% (n = 10/312) reporting being unsure of the method most commonly used. When asked about tools commonly used for mechanical methods (Figure 3), 32.5% (n = 103/312) were unsure of the tools used, followed by using metal rods (27.1%; n = 86/312), closed scissors (20.2%; n = 64/312) and pens (15.8%; n = 50/312). Some participants (7.9%; n = 25/312) also listed several ‘other’ tools which included, but were not limited to, card cage holders, tweezers, closed forceps, cage dividers, and plastic or metal scrapers (for full list, see Supplementary material). A few participants also reported using haemostats (2.5%; n = 8/312) and rulers (1.9%, n = 6/312). We found no effect of an individual’s role, sector or experience level on their likelihood of using manual or mechanical methods, nor their choice of tool.

Participants were also asked whether the mouse was restrained on a flat surface, such as a cage lid, when performing cervical dislocation. We found that 91.0% (n = 284/312) of participants said yes, 6.7% (n = 21/312) responded no, and 2.2% (n = 7/312) stated they did not know. In addition, participants were also asked whether their institution had a dedicated Standard Operating Procedure (SOP) detailing the preferred technique for cervical dislocation of laboratory mice. We found that 63.5% (n = 198/312) of respondents said yes, 14.4% (n = 45/312) said no, and 22.1% (n = 69/312) stated they did not know.

### Criteria considered important for successful cervical dislocation of mice

**Figure 4.**
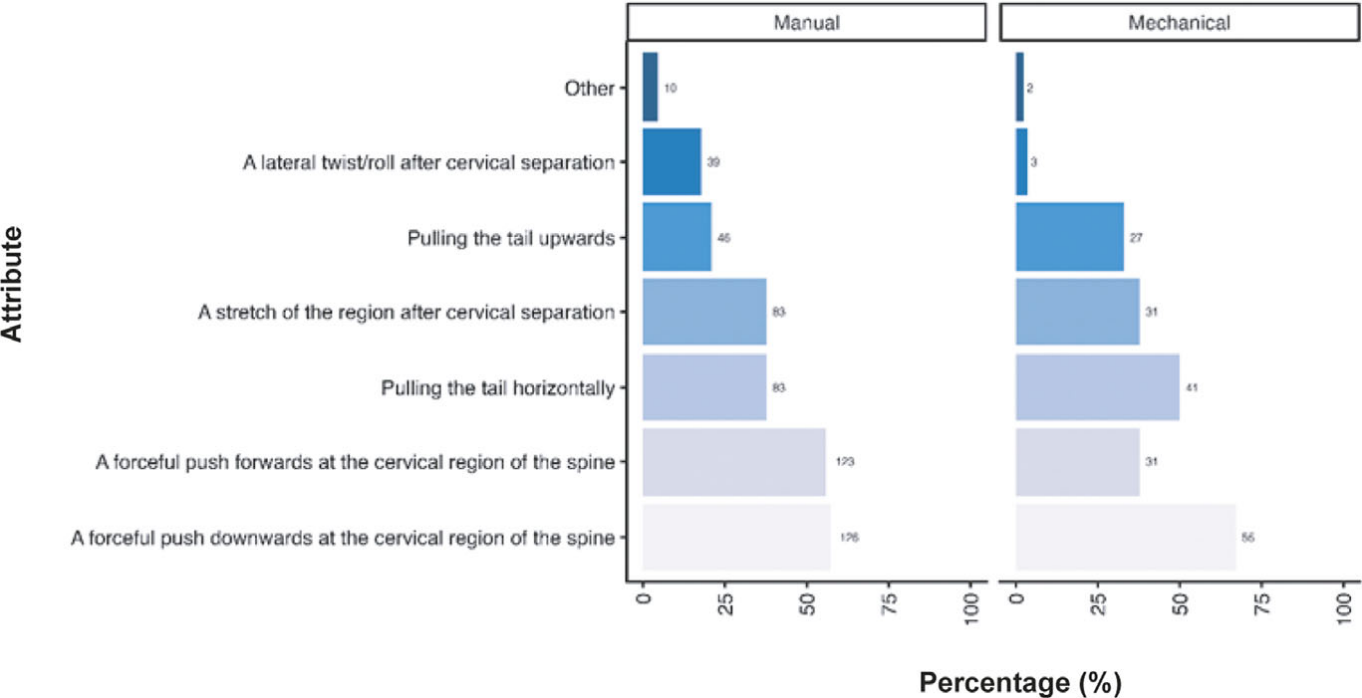
Percentage (%) of participants working with laboratory mice (*Mus musculus*; total; n = 312) according to the importance of each criteria for successful cervical dislocation of mice. Calculated separately for those primarily utilising manual vs mechanical methods (manual total; n = 220, mechanical total; n = 82).

Participants were also asked their opinion regarding the criteria they considered necessary for successful cervical dislocation in mice. A similar pattern of responses was observed across respondents, irrespective of them primarily using manual methods (total; n = 220) and mechanical methods (total; n = 82), although some notable differences were observed (Figure 4). Most participants considered a forceful push downwards of the cervical region of the spine the most important criterion required for successful cervical dislocation (manual: 57.3%; n = 126/220, mechanical: 67.1%; n = 55/82). This was closely followed by a forceful push forward at the cervical region of the spine for manual methods (55.9%; n = 123/220), yet pulling the tail horizontally was deemed necessary by more individuals when utilising mechanical methods (50.0%; n = 41/82) compared to those using manual methods (37.7%; n = 83/220). A stretch of the region following cervical separation was deemed important for over one-third of participants for both methods (manual: 37.7%; n = 83/220, mechanical: 37.8%; n = 31/82). For those using mechanical methods, pulling the tail upwards was considered important for success in approximately one-third of respondents (32.9%; n = 27/82), which was not the case for manual methods (20.9%; n = 46/220). A lateral twist/roll after cervical separation was considered more important in individuals utilising manual methods (17.7%; n = 39/220) compared to those utilising mechanical methods (3.7%; n = 3/82). A few respondents provided other criteria (manual: 4.5%; n = 10/220, mechanical: 2.4%; n = 2/82) they considered important, which are included in the Supplementary material. A summary of the combinations of answers for the attributes considered important for successful cervical dislocation in mice, according to the primary method (manual or mechanical), is also included in Table S1 (Supplementary material).

**Figure 5.**
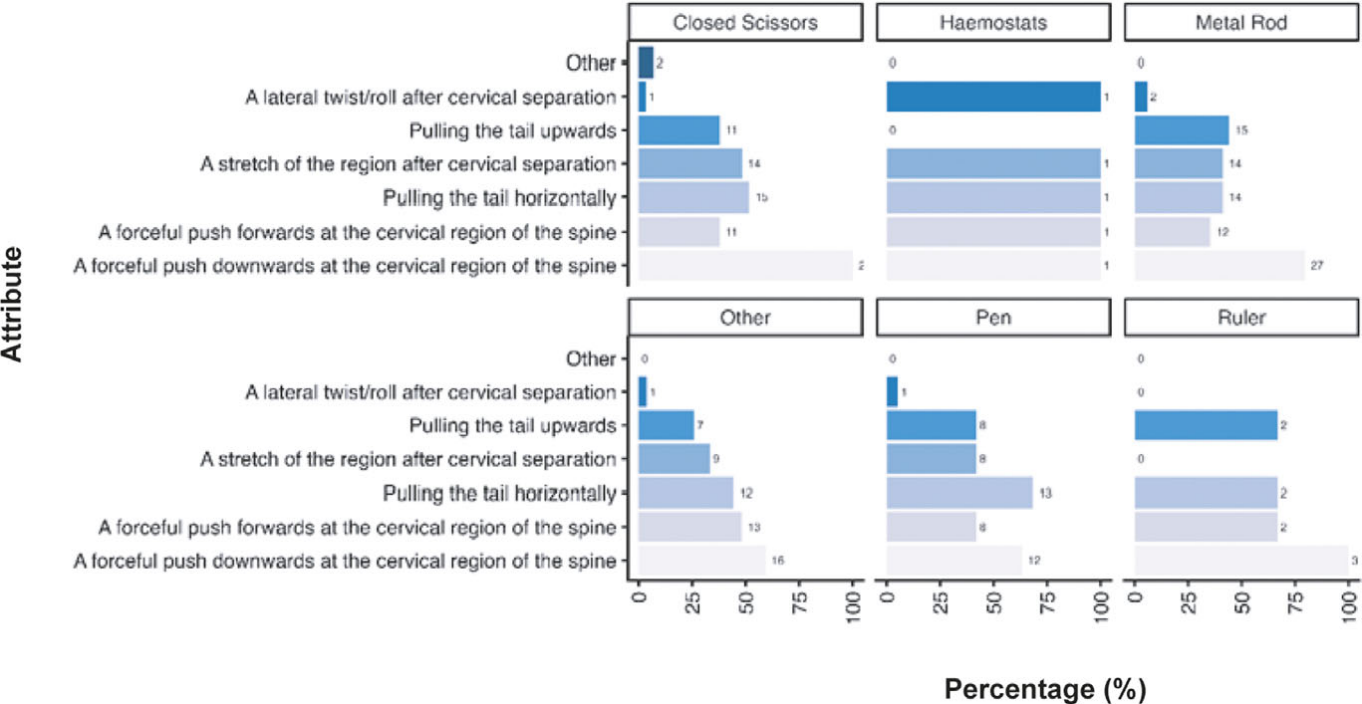
Percentage (%) of participants working with laboratory mice (*Mus musculus*; total; n =312) according to the importance of each criteria for successful cervical dislocation of mice. Calculated separately according to individual tool use for those primarily utilising mechanical methods (mechanical total; n = 82, pen; n = 19/82, metal rod; n =34/82, closed scissors; n = 29/82, haemostats; n = 1/82, ruler; n = 3/82, other; n = 27/82).

Given the diversity in tool use across the sector for mechanical methods we also examined the criteria participants considered necessary for the cervical dislocation of mice according to tool (Figure 5). As outlined previously, a forceful push downwards at the cervical region of the spine was considered important across all tools (closed scissors: 100%; n = 29/29, haemostats: 100%; n = 1/1, metal rod: 79.4%; n = 27/34, pen: 63.2%; n =12/19, ruler: 100%; n = 3/3, other: 59.3%; n = 16/27) however for those utilising pens pulling the tail horizontally was considered equally as important (68.4%; n = 13/19). Only small numbers or participants predominantly utilising mechanical methods for laboratory mice used haemostats (n = 1) and rulers (n = 3). As outlined previously, participants describing other tools (n = 27) included, but were not limited to, card cage holders, tweezers, closed forceps, cage dividers, and plastic or metal scrapers (for full list, see Supplementary material).

Participants were asked how humane they considered cervical dislocation for mice (Table 2). The majority of respondents (87.4% of all respondents and 87.5% of those working with mice) moderately or strongly agreed with the statement that ‘cervical dislocation is a humane method of euthanasia for laboratory mice’. However, participants were more likely to consider cervical dislocation a humane technique for laboratory mice if they stated using manual methods compared to those utilising mechanical methods (X_2_^[2]^ = 344.9; *P* < 0.001).

**Table 2.** Summary of participant responses for the question asking ‘to what extent do you agree with the following statement “cervical dislocation is a humane method of euthanasia for laboratory mice”. The number and percentage of all respondents (n = 317) and the number and percentage of respondents working with mice (n = 312)

Answer	Number of all respondents (n = 317)	Percentage of all respondents (%)	Number of respondents working with mice (n = 312)	Percentage of respondents working with mice (%)
Strongly agree	166	52.4	164	52.6
Moderately agree	111	35.0	109	34.9
Slightly agree	21	6.6	21	6.7
Slightly disagree	9	2.8	8	2.6
Moderately disagree	5	1.6	5	1.6
Strongly disagree	4	1.3	4	1.3
Unknown	1	0.3	1	0.3

### Cervical dislocation techniques for laboratory rats

**Figure 6.**
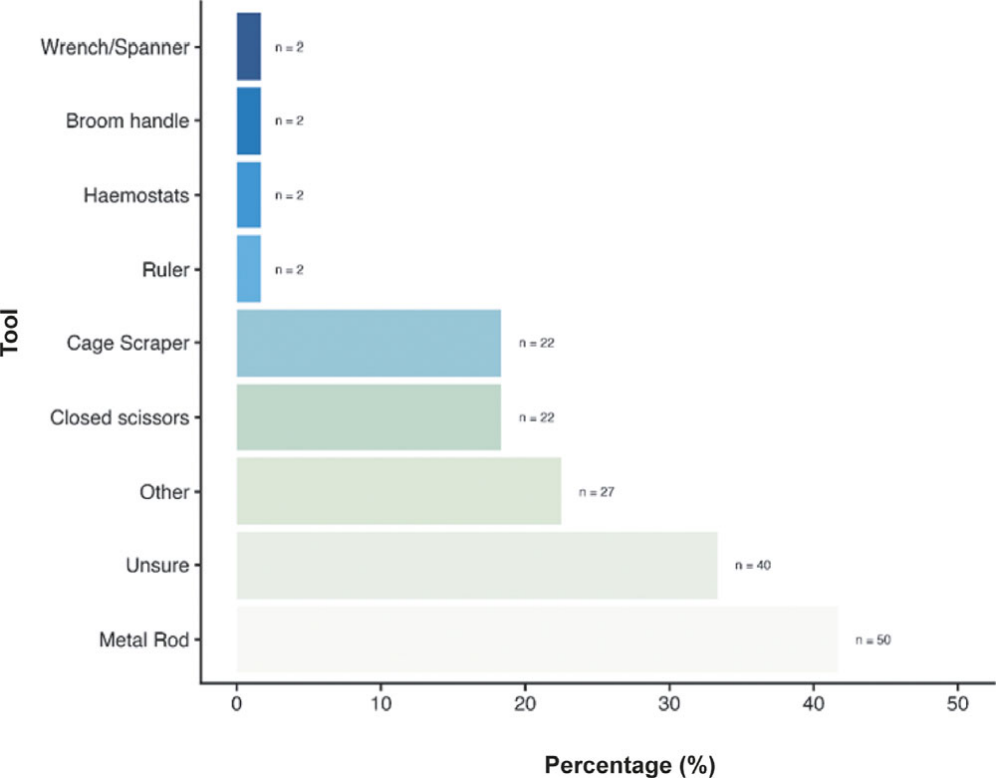
Percentage (%) of tools commonly used for cervical dislocation of those working with laboratory rats (*Rattus norvegicus*; total; n = 120).

We also asked about the most common techniques used for cervical dislocation for those working with laboratory rats. N = 81/120 (67.5%) reported that mechanical methods (e.g. using dedicated equipment or tool) were primarily used, with only 15.0% (n = 18/120) reporting using manual methods, and 17.5% (n = 21/120) reporting they were unsure of the method most frequently used at their institution. When asked about tools commonly used for mechanical cervical dislocation of rats (Figure 6), 41.7% (n = 50/120) stated that metal rods were most used, with 33.3% (n = 40/120) not being sure what tools were commonly used. N = 27/120 (22.5%) of participants listed several ‘other’ tools including but not limited to, card cage scrapers, plastic scrapers, cage water bottle dividers (for full list, see Supplementary material). Participants also reported using closed scissors (18.3%; 22/120) and cage scrapers (18.3%; 22/120) and a few reported using haemostats (1.7%; n = 2/120), rulers (11.7%; n = 2/120), broom handles (1.7%; n = 2/120) and wrenches or spanners (1.7%; n = 2/120). We found no effect of an individual’s role, sector or experience level on their likelihood to use manual or mechanical methods nor their choice of tool.

Participants were asked whether the rat was restrained on a flat surface when performing cervical dislocation. N = 105/120 (87.5%) of participants said yes, 3.3% (n = 4/120) responded no, and 9.2% (n = 11/120) stated they did not know. In addition, participants were also asked whether their institution had a dedicated Standard Operating Procedure (SOP) detailing the preferred technique for cervical dislocation of laboratory rats. N = 75/120 (62.5%) of respondents said yes, 27.5% (n = 33/120) said no, and 10.0% (n = 12/120) stated they did not know.

### Criteria considered important for successful cervical dislocation of rats

**Figure 7.**
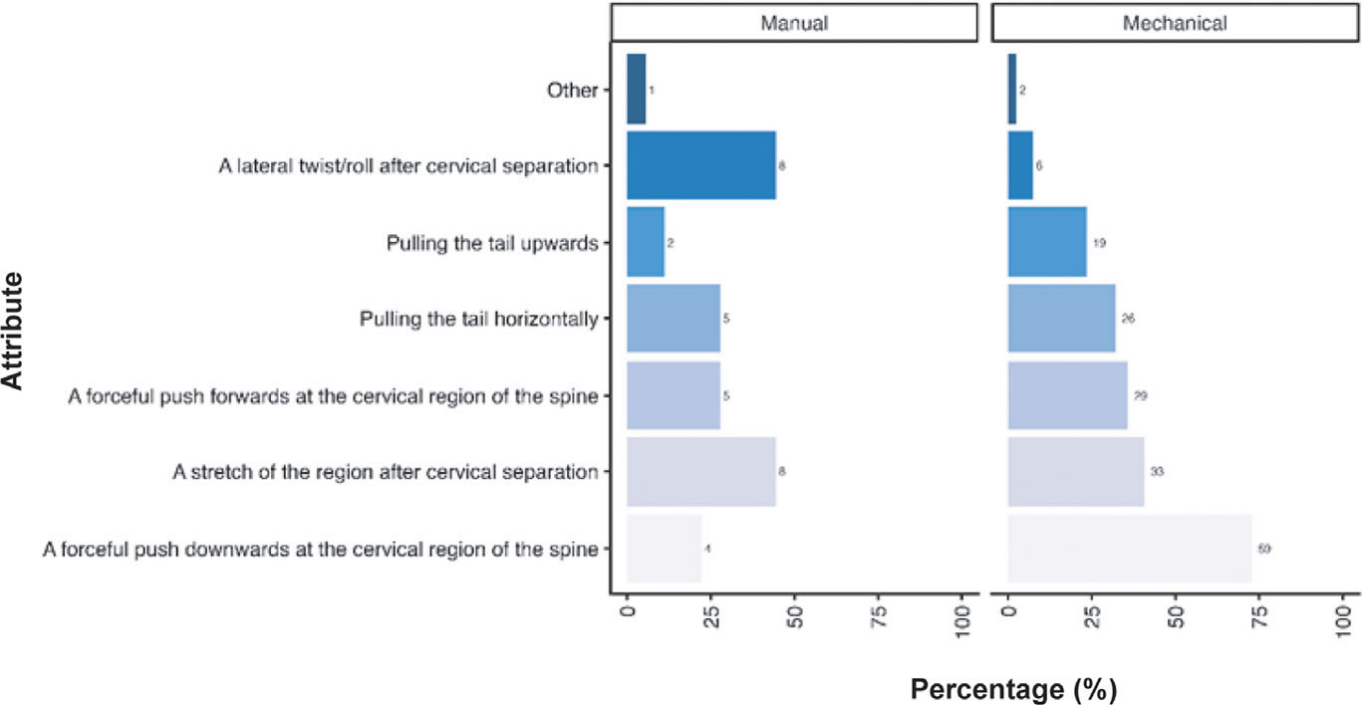
Percentage (%) of participants working with laboratory rats (*Rattus norvegicus*; total; n = 120) according to the importance of each criteria for successful cervical dislocation of rats. Calculated separately for those primarily utilising manual versus mechanical methods (manual total; n = 18/120, mechanical; n = 81/120).

As with the questions asked of laboratory mice, participants were asked for their opinions on the criteria they considered necessary for successful cervical dislocation in rats. The criteria considered most important were different according to the method (manual vs mechanical) participants stated they primarily used (Figure 7). For those primarily using manual methods (total n = 18), the two criteria considered necessary for the successful cervical dislocation were a lateral twist/roll (44.4%; n = 8/18) or stretch of the region or after cervical separation (44.4%; n = 8/18). Whereas pulling the tail horizontally (27.8%, n = 5/18), a forceful push forwards (27.8%; n = 5/18) or downwards (22.2%; n = 4/18) in the cervical region and pulling the tail upwards (11.1%; n = 2/18) were considered necessary by less than one-third of participants. For those primarily using mechanical methods (n = 81), they considered a forceful push downwards in the cervical region of the spine (72.8%; n = 59/81) and a stretch of the region after cervical separation (40.7%; n = 33/81) the two most important properties for effective dislocation. N = 29/81 (35.8%) stated a forceful push forward was necessary compared to 32.1% (n = 26/81) pulling the tail horizontally. Fewer participants considered pulling the tail upwards (23.5%; n = 19/81) and a lateral twist/roll (7.4%; n = 6/81) after cervical separation as necessary. A few respondents provided other criteria (manual: 5.6%; n = 1/18, mechanical: 2.5%; n = 2/8) they considered necessary which are included in the Supplementary material.

**Figure 8.**
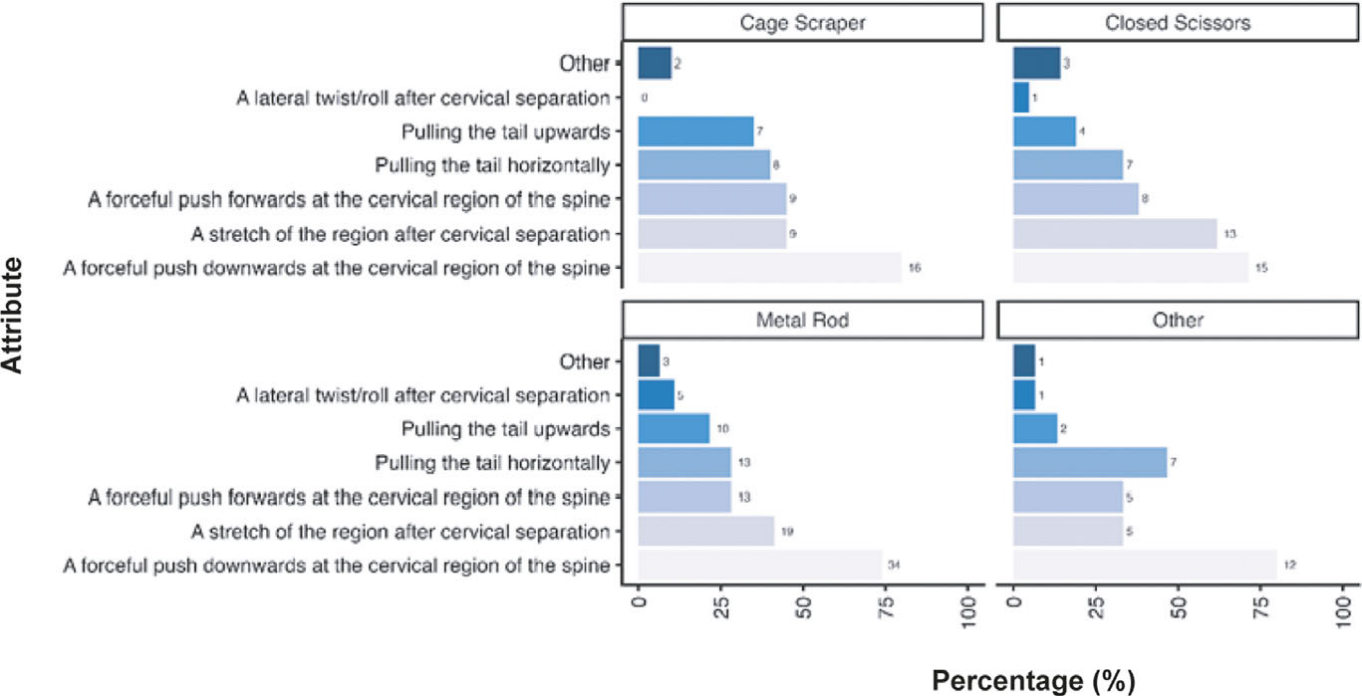
Percentage (%) of participants working with laboratory rats (*Rattus norvegicus*; total n = 120) according to the importance of each criteria for successful cervical dislocation of rats. Calculated separately according to individual tool use for those primarily utilising mechanical methods (mechanical total; n = 81, cage scraper; n = 20/81, metal rod; n = 46/81, closed scissors; n = 21/81, other; n = 15/81).

We also examined how participants considered these criteria necessary according to tool (Figure 8). A forceful push downwards at the cervical region of the spine was considered important across all tools (closed scissors: 71.4%; n = 15/21, metal rod: 73.9%; n= 34/46, cage scraper: 80.0%; n = 16/20, other: 80.0%; n = 12/15). Only small numbers of participants predominantly utilising mechanical methods for laboratory rats stated that they used haemostats (n = 2), rulers (n = 2), broom handles (n = 2), and wrenches or spanners (n = 2). As outlined previously, participants describing other tools (n = 40) included, but were not limited to, card cage holders, cage dividers, plastic or metal scrapers, dedicated cervical dislocators (either commercially available or made in-house) (for full list, see Supplementary material).

Participants were asked how humane they considered cervical dislocation for rats (Table 3). When considering the responses from all participants, the majority of respondents (36.9%) responded ‘unknown’ to the statement ‘cervical dislocation is a humane method of euthanasia for laboratory rats’. However, when considering the responses from those who stated working with rats, approximately half of the respondents stated they moderately (29.2%) or strongly agreed (21.7%) with the statement ‘cervical dislocation is a humane method of euthanasia for laboratory rats’. We found no effect of the primary method (manual or mechanical), sector, role or experience of participants on how humane they consider cervical dislocation for laboratory rats.

**Table 3.** Summary of participant responses for the question asking ‘to what extent do you agree with the following statement “cervical dislocation is a humane method of euthanasia for laboratory rats”. The number and percentage of all respondents (n = 317) and the number and percentage of respondents working with rats (n = 120)

Answer	Number of all respondents (n = 317)	Percentage of all respondents (%)	Number of respondents working with rats (n = 120)	Percentage of respondents working with rats (%)
Strongly agree	54	17.0	26	21.7
Moderately agree	58	18.3	35	29.2
Slightly agree	35	11.0	18	15.0
Slightly disagree	15	4.7	10	8.3
Moderately disagree	20	6.3	14	11.7
Strongly disagree	18	5.7	11	9.2
Unknown	117	36.9	6	5.0

## Discussion

This study provides the first detailed insight into how cervical dislocation of laboratory rodents is currently performed across UK research institutions. We found marked inconsistencies in both technique and the use of tools to aid application, with mice predominantly killed via manual operation (i.e. without the aid of tool), while rats were more often killed using mechanical aids. A wide range of improvised tools (i.e. not designed for the purpose of killing) were reported, including pens, scissors, and cage scrapers. Across all laboratory rodents, there was little or no consensus regarding which physical actions are essential for a successful dislocation (i.e. the necessity of a stretch and/or a twist), or in institutional standard operating procedures (SOPs).

One of the most striking results from this study was the divergence in approach between mice and rats. Most participants reported relying on manual operation solely for mice (manual 69.5% vs mechanical aid 25.9%), whereas mechanical tools dominated the killing of rats (manual 15.0% vs mechanical aid 67.5%). This division is not reflected in current legislation, where rodents (i.e. both rats and mice) are grouped together under Schedule 1 of the Animals (Scientific Procedures) Act 1986) (Home Office 1986). The Act permits cervical dislocation up to 500 g for rodents, but imposes additional requirements for rodents over 150 g, such as the use of anaesthesia or sedation. Although mice are unaffected by these weight thresholds due to their smaller size (with typical adult bodyweights rarely exceeding 40 g [Home Office 2014]), typical laboratory rats quickly exceed 150 g (Home Office 2014), meaning the use of a sedative or anaesthetic are often legally required when applied to adult rats. However, the Act does not distinguish between manual or mechanically aided cervical dislocation. This differs from agricultural legislative regulation for the protection of livestock. Under the Welfare of Animals at the Time of Killing (England) Regulations (WATOK) (UK Government 2015), cervical dislocation is permitted in poultry up to 5 kg, however the regulation distinguishes between manual and mechanically operated cervical dislocation, and manual operation is further restricted by a lower bodyweight limit (< 3 kg). Importantly, unlike in rodents, attention and scientific research into the effectiveness and humaneness of cervical dislocation of poultry (both manual and mechanical methods) has been subject to scientific interest since 2010, with limited research in other species. It is notable that the scientific basis and motivational drivers for weight restrictions for rodent cervical dislocation are unclear. It can be inferred that there may be potential welfare impacts or operational challenges when cervical dislocation is applied to conscious rodents weighing greater than 150 g, hence requiring the animal to be anaesthetised (i.e. fully unconscious) to negate potential negative animal welfare impacts. The use of sedation (i.e. drug-induced depression of consciousness, ranging from minimal to moderate (Blayney 2012)), could also be an attempt to alleviate potential animal welfare harms, but may also act as a method to overcome challenges in handling and restraining the animal to apply cervical dislocation effectively (i.e. inducing a relaxed more compliant state in the animal (Blayney 2012)). To our knowledge, there is no direct evidence directly comparing the ease and accuracy of cervical dislocation between species to justify the weight restrictions, in addition to the scientific evidence base on which these thresholds originated. Anecdotally, there are examples of practice guidance advising that rodents weighing greater than 150 g should not be subject to cervical dislocation due to heavier cervical musculature (e.g. Leary *et al.* 2020). However, this information is not evidenced within the guidance itself, and refers to a further guidance document relating to wild bird research (Fair *et al.* 1997) and there are no published scientific data showing increased cervical musculature as negatively affecting successful cervical dislocation in rats. Therefore it is possible that these legislative restrictions, may be inadvertently driving a greater dependence on improvised mechanical tools/aids for rats. In contrast, the predominance of manual techniques for mice could reflect both the absence of weight-related constraints and a perception that manual techniques are more immediate and reliable (Clarkson *et al.* 2022). Importantly, our findings suggest that differences in method between rats and mice, may be driven as much by regulatory thresholds and institutional culture as by (limited) scientific evidence. There is a need to re-examine the rationale for existing weight limits and to establish whether they genuinely reflect differences in effectiveness or welfare outcomes. Without such evidence, there is a risk that regulatory frameworks unintentionally promote the use of unvalidated mechanical methods, particularly in rats. This would potentially be to the detriment of animal welfare (e.g. risk of crushing/asphyxiation and/or unnecessary suffering) reported for a range of methods/techniques available for poultry (Sparrey *et al.* 2014).

Fundamentally, cervical dislocation, whether applied manually or with the use of a mechanical aid, aims to cause intervertebral cervical separation of the vertebral column, causing extensive damage to the spinal cord and surrounding tissues, whilst disrupting blood flow to the brain (ideally through damage/severance of the carotid arteries in the neck) resulting in death via cerebral ischaemia (Cartner *et al.* 2007; Suckow *et al.* 2012; Clarkson *et al.* 2022). Whilst this mechanism for inducing death via cervical dislocation is broadly agreed across the scientific community, the required actions (i.e. physical manipulation of the neck etc.) are far from established. As there is little or no scientific literature focusing on these actions in rodents, evidence from other species could provide valuable insight. In poultry, there is general agreement that cervical dislocation should involve a rapid stretch and twist action, resulting in more extensive damage to the neck, spinal cord and surrounding tissues and rapid disruption of blood flow to the brain, leading to a faster onset of unconsciousness and death (Mikeska & Klemm 1975; Gregory & Wotton 1990; Cartner *et al.* 2007; Carbone 2011; Kongara *et al.* 2014). In comparison, cervical dislocation which only involved one component (i.e. stretch or twist alone), resulted in less extensive trauma, extended onset to unconsciousness and is therefore considered less humane with animal welfare outcomes similarly poor to those observed for decapitation (Mikeska & Klemm 1975; Gregory & Wotton 1986; van Rijn *et al.* 2011; Martin *et al.* 2019). As highlighted by the results of this survey, there is a broad range in techniques and tools/aids used to cause cervical dislocation in both rats and mice (see Figures 3–8). For both rats and mice, in the case of mechanical cervical dislocation, over 50% of respondents expressed a technical need to apply “a forceful push downwards at the cervical region of the spine”, irrespective of the tool/aid used. Based on evidence from other species (e.g. Gregory & Wotton 1990; Holson 1992; Close *et al.* 1997; Bates 2010; Erasmus *et al.* 2010, Martin *et al.* 2016), this poses a potential animal welfare concern, as such an action alone could be considered more akin to crushing the neck and more likely to cause prolonged suffering through delayed onset of unconsciosuness as well as death via asphyxia. As such, any technique considered to primarily cause crushing injury to induce cervical dislocation is prohibited for poultry in agricultural settings in both Great Britain and Europe (European Council [EC] 2009; UK Government 2015), highlighting the greater risk of suffering if applied to conscious animals, including rodents. In contrast, neither regulation governing the killing of animals used for scientific purposes in Great Britain or Europe refer to crushing risks, nor define the mode of action for cervical dislocation in any species for which cervical dislocation is permitted (Home Office 1986; European Parliament and the Council of the European Union 2010). This survey, along with other recent communications (Davidge *et al.* 2025; Ferreira 2025), highlights the requirement for scientific exploration relevant to laboratory rodents to encourage potential reform to existing guidance and legislation.

A striking finding of this survey was the widespread reliance on improvised tools for mechanical cervical dislocation in both mice and rats. Respondents listed items such as pens, scissors, metal rods, cage scrapers and even spanners — none of which have been designed, validated, or standardised for the purpose of killing animals. This survey did not explore the reasons for specific improvised tools being used or selected. This widespread reliance may indicate attempts to refine killing practices at a local level. To our knowledge, there are currently no commercially available, purpose-designed tools for rodent cervical dislocation which have been scientifically validated as ‘humane’, despite the method being both common and widely permitted under legislation (Clarkson *et al.* 2022). The use of improvised tools, such as those identified in this survey, raises serious potential welfare concerns. Firstly, these items are not engineered to apply force in a controlled or consistent manner, meaning that the risk of inaccurate dislocation (e.g. location of dislocation, incomplete/partial dislocation, crushing injury etc) is substantial (Cartner *et al.* 2007; Carbone *et al.* 2012). Additionally, as these tools were not designed with cervical dislocation in mind, the tools themselves may compromise execution of the technique, for example, pens may break during use, scissors may slip, and blunt rods or scrapers may induce crush injuries rather than dislocation. This increases the likelihood of thoracic as opposed to cervical separation, which has been documented in previous studies (Cartner *et al.* 2007; Carbone *et al.* 2012) and is likely to prolong the time to unconsciousness and death (as denoted in poultry; Martin *et al.* 2019). The shape and size of tools used to apply cervical dislocation is likely to be a critical component of their efficacy and humaneness. Relying on scientific data from poultry as none exists for rodents, the size of the ‘edge’ employed to push down on the neck to provide the force required to induce intervertebral dislocations, is essential, and variability in tool performance was linked to age/size of birds, as well as operator technique (Erasmus *et al.* 2010; Martin *et al.* 2017, 2018a,b; Woolcott *et al.* 2018; Bandara *et al.* 2023; Ripplinger *et al.* 2024). Given the optimal application of cervical dislocation requires intervertebral dislocation at either the atlanto-occipital joint (C0–C1) or the atlanto-axial joint (C1–C2) (Carbone *et al.* 2012), being able to target such locations to apply the tool is essential. In mice, the cervical vertebrae are < 1 mm in diameter, typically with the area from the base of the skull to C3 being < 2.5 mm, therefore, any ‘edge’ with a diameter of greater than 1 mm is unlikely to result in intervertebral cervical dislocations above vertebra C3 whilst avoiding unnecessary crushing injury (Babak Kalantar 2013; Smorgick & Fischgrund 2013; Bader *et al.* 2014; Martin *et al.* 2017, 2018b; Bandara *et al.* 2023; Nkurikiyumukiza *et al.* 2024). Secondly, manual and many of the mechanical techniques using these improvised tools may rely on additional actions, such as tail pulling, introducing further failure risks, as well as the potential for unnecessary injuries to the animal (e.g. direct injuries to the tail from pulling (Machholz *et al.* 2012; Gencturk & Unal 2024; Davidge *et al.* 2025)).

This survey also revealed a lack of consensus regarding which additional physical actions are necessary for a successful cervical dislocation. Manual operators typically emphasised a strong push forward or downward, with some citing a lateral twist, whereas operators who utilised a tool/aid (i.e. mechanical) more often stressed a downward push combined with pulling the tail. These divergent views suggest that current practice is highly driven at the institutional level, relying on ‘local processes’ and personal belief/preferences rather than empirical evidence. This was further supported in this survey through highlighting the inconsistent availability and content of SOPs both within and between UK institutions. For mice, over one in five respondents did not know whether an SOP existed at their institution, while for rats, nearly 30% stated that no SOP was in place. These findings suggest a lack of institutional clarity and standardisation, despite cervical dislocation being one of the most common killing methods used in the UK (Clarkson *et al.* 2023). Previous studies have shown that dislocation frequently occurs at thoracic rather than cervical levels in mice (Cartner *et al.* 2007; Carbone 2011), but they did not describe the cervical dislocation technique in sufficient detail and/or specific additional actions used. It therefore remains unknown which techniques most reliably achieve high cervical dislocation and rapid unconsciousness in mice.

Our findings highlight a critical knowledge gap: systematic, controlled studies are needed to determine which techniques reliably achieve humane outcomes, and whether different approaches carry risks of unnecessary injury, such as tail damage or thoracic trauma and/or dislocation. The reliance on improvised tools highlights an important gap in knowledge in the required physical actions for humane and effective dislocation in rodents (e.g. stretch and/or twist), needed to inform tool design. Without purpose-built, validated devices/tools, research institutions are potentially placing both personnel and animals in a vulnerable position and inadvertently not aligning with the legal requirement to minimise unnecessary pain, distress and suffering (Home Office 1986). From an ethical standpoint, it is questionable whether it is acceptable for research institutions to sanction the use of equipment not designed for killing, particularly when there are serious potential animal welfare consequences. This finding underscores the urgent need for research into the design, validation, and implementation of dedicated cervical dislocation tools for rodents that are both effective and ergonomic for the user.

Finally, as a self-report survey, this study captures perceptions rather than direct observations of practice. Some questions were answered by individuals who may not routinely perform cervical dislocation on rats, potentially impacting the summarised perceptions reported. In addition, responses were anonymous, meaning that representativeness of the UK sector could not be verified. However, anonymity was essential to encourage responder engagement, given the subject matter is sensitive in nature and the use of animals for scientific purposes is under constant scrutiny from both the public and media. Nevertheless, the survey achieved a large sample size (n = 317) across the UK, across diverse roles and institutions, providing valuable insights into sector-wide practices and uncertainties.

### Animal welfare implications

This survey highlights the potential welfare risks in the current use of cervical dislocation to kill laboratory rodents. Practices vary widely across institutions, with many relying on improvised tools such as pens or scissors that were never designed for this purpose. Such inconsistency raises the likelihood of inaccurate dislocation, delayed loss of consciousness, and unnecessary suffering. The lack of evidence-based guidance, purpose-built tools, and standardised training compounds these risks. Urgent action is needed to establish validated methods and clear standards to ensure this common killing technique is applied humanely and consistently.

## Conclusion

Our findings highlight a clear need for further research and potential reform in current practice. Empirical studies are required to determine which techniques and physical actions consistently achieve rapid unconsciousness and death in rodents when using cervical dislocation. The development and validation of purpose-designed tools is also essential to replace the ongoing reliance on improvised devices. Additonally, regulatory thresholds relating to weight limits set out in guidance and legislation, warrant further examination in light of current scientific evidence and comparison with agricultural standards. Finally, the establishment of clear, standardised SOPs, supported by structured training, is crucial to reduce variability and improve consistency in practice. In conclusion, cervical dislocation remains a common method for killing laboratory rodents in the UK, yet this survey reveals an inconsistency in its application. The absence of dedicated tools, uncertainty over the most effective technique, and uneven implementation of SOPs present ongoing risks to animal welfare. Without urgent progress towards evidence-based, standardised approaches, rodents will continue to be killed by methods that are both potentially unreliable and ethically problematic.

## Supporting information

10.1017/awf.2026.10087.sm001 Martin et al. supplementary material Martin et al. supplementary material

## Supplementary material

The supplementary material for this article can be found at http://doi.org/10.1017/awf.2026.10087. All data and respective analyses files are freely available at http://doi.org/10.5281/zenodo.19677806.
