# Supplementary material for: Inconsistency in cervical dislocation: A UK survey of techniques and tools utilised for laboratory rodents
Source: Anim Welf. 2026 Apr 29;35:e32. doi: 10.1017/awf.2026.10087 (PMC13126198; doi:10.1017/awf.2026.10087)
Supplement: Martin et al. supplementary material [file S0962728626100876sup001.pdf]

# Inconsistency in cervical dislocation: A UK survey of techniques and tools utilised for laboratory rodents: Supplementary materials

Jessica E Martin[1], Matthew C Leach[2], Jasmine M Clarkson https://orcid.org/0000-0001-5899-4245[1]

[1] School of Natural and Environmental Sciences, Newcastle University

[2] Comparative Biology Centre, Newcastle University

Author for correspondence: Jasmine M Clarkson, email: jasmine.clarkson@newcastle.ac.uk

# Methods and techniques used for the cervical dislocation of laboratory rodents.

The aim of this study is to determine what techniques are used for cervical dislocation of laboratory rodents across UK.

We invite you to complete a very short questionnaire (~5mins). The research is being conducted as part of a wider project investigating potential alternative, humane methodologies for rodent euthanasia. To participate you must be over 18 and work for an established research institution (e.g., academia or industry) and have experience working with laboratory rodents.

Completion of the survey is entirely voluntary and anonymous, and we will not collect any identifiable information from you for more information about data processing see (hyperlink to privacy notice). Nevertheless, all study data will be held in accordance with The General Data Protection Regulation (2018). The data will be stored in archiving facilities in line with Newcastle University's retention policy of up to 10 years (add link).

This study has been approved by Newcastle University's Animal Welfare and Ethical Review Body.

Contact for further information: Dr Jasmine Clarkson (jasmine.clarkson@newcastle.ac.uk)

**Consent**

I confirm consent to the following:

- I confirm that I have read and understood the information provided
- I understand that all data and information I provide will be anonymous and will be seen only by study researchers
- I confirm that I agree that data will be stored for at least 10 years in the University archiving facilities in accordance with relevant Data Protection policies and regulations.
- I understand that if I withdraw from the study, my data collected up to that point may be retained and used for the remainder of the study.

I confirm that I consent to take part in the study. ☐

## Part 1: Demographics:

1. Which sector(s) do you currently work in?
    a. Academic
    b. Pharmaceutical
    c. Contract Research Organisation
    d. Other (please specify)

2. What best describes your current role?
    a. Technician
    b. Veterinarian
    c. Researcher
    d. Regulatory
    e. Other (please specify)

3. How many years' experience, do you have working with laboratory rodents?
    a. 0-1 years
    b. 2-5 years
    c. 6-11 years
    d. 12-17 years
    e. 18-23 years
    f. 24 + years

4. What laboratory species do you work with? (select all that apply)
    a. Mice
    b. Rats
    c. Hamsters
    d. Gerbils
    e. Guinea pigs
    f. Other (please specify)

## Part 2: Techniques used for cervical dislocation of laboratory mice:

5. What is the most common technique for the cervical dislocation of laboratory **mice** at your research institution.
    a. Manual using just fingers (this does not involve the use of any dedicated tool/equipment to separate the cervical vertebrae)
    b. Mechanical (this describes methods that may utilise dedicated tools to separate the vertebrae).

6. If mechanical techniques are commonly used, what tool/equipment is utilised? (select all that apply)
    a. Pen
    b. Ruler
    c. Metal rod
    d. Closed scissors
    e. Haemostats
    f. Other
        i. Please specify [free text option]

7. If manual techniques are commonly used, is the mouse restrained on a flat surface (e.g., such as a cage lid) to perform the dislocation?
    a. Yes
    b. No

8. If the mouse is not restrained on a flat surface, what best describes the application of the technique? (only appears to those who answer No to Q7)
    a. The mouse is restrained via the scruff in an upright position and the technique is applied by the free hand.
    b. The mouse is gently restrained in the hand and the technique is applied by the thumb and forefinger.
    c. Other
        i. Please specify [free text option]

9. In your opinion, does the technique required for successful cervical dislocation of mice rely on any of the following: (select all that apply)
    a. Pulling the tail horizontally
    b. Pulling the tail upwards
    c. A forceful push forwards at the cervical region of the spine
    d. A forceful push downwards at the cervical region of the spine
    e. A lateral twist/roll after cervical separation
    f. A stretch of the region after cervical separation
    g. Other (please specify) [free text option]

10. Is there a dedicated Standard Operating Procedure (SOP) at your research institution, detailing the preferred technique for the cervical dislocation of laboratory mice?
    a. Yes
    b. No
    c. Don’t know

11. In your opinion, to what extent do you agree with the following statement “cervical dislocation is a humane method of euthanasia for laboratory mice”?

    ☐Strongly disagree ☐ Moderately Disagree ☐ Slightly disagree ☐ Slightly Agree ☐ Moderately Agree ☐Strongly Agree ☐Unknown

## Part 2: Techniques used for cervical dislocation of laboratory rats:

12. What is most commonly used technique in your research institution for the cervical dislocation of laboratory **rats**.
    a. Manual using fingers (this does not involve the use of any dedicated tool/equipment to separate the cervical vertebrae)
    b. Mechanical (this describes methods that may utilise dedicated tools to separate the vertebrae).

13. If mechanical techniques are commonly used, what tool/equipment is utilised? (select all that apply)
    a. Ruler
    b. Metal rod
    c. Closed scissors
    d. Haemostats
    e. Cage scraper
    f. Broom handle
    g. Wrench/spanner
    h. Other
        i. Please specify [free text option]

14. If manual techniques are commonly used, is the rat restrained on a flat surface (e.g., such as a cage lid) to perform the dislocation?
    a. Yes
    b. No

15. If the rat is not restrained on a flat surface, what best describes the application of the technique? (only appears to those who answer No to Q12)
    a. The rat is restrained via the scruff in an upright position and the technique is applied by the free hand.
    b. The rat is gently restrained in the hand and the technique is applied by the thumb and forefinger.
    c. Other
        i. Please specify [free text option]

16. Does the chosen technique differ according to prior sedation and the weight of the rat?
    a. Yes
    b. No
    c. Don't know

17. In your opinion, does the technique required for successful cervical dislocation of rats rely on any of the following: (select all that apply)
    a. Pulling the tail horizontally
    b. Pulling the tail upwards
    c. A forceful push forwards at the cervical region of the spine

d. A forceful push downwards at the cervical region of the spine
e. A lateral twist/roll after cervical separation
f. A stretch of the region after cervical separation
g. Other (please specify) [free text option]

18. Is there a dedicated Standard Operating Procedure (SOP) at your research institution, detailing the preferred technique for the cervical dislocation of laboratory rats?
    a. Yes
    b. No
    c. Don't know

19. In your opinion, to what extent do you agree with the following statement "cervical dislocation is a humane method of euthanasia for laboratory rats"?

    ☐Strongly disagree ☐ Moderately Disagree ☐ Slightly disagree ☐ Slightly Agree ☐ Moderately Agree ☐Strongly Agree ☐Unknown

## Thank You Page

You have now completed this survey and your responses have been recorded.

If you have any further questions about this study, please do not hesitate to contact Dr Jasmine Clarkson via jasmine.clarkson@newcastle.ac.uk.

Thank you, your participation in this research is greatly appreciated.

**<u>Q6 Cervical dislocation techniques for laboratory mice</u>**

**'Other' tools responses included:**

Cage card holder

Tweezers

Mechanical not used. Could not leave question blank

closed forceps

plastic scraper

Ice scraper

Car ice scraper

plastic paint scraper like tool

PLASTIC SCRAPER

Wall paper scraper (blunted)

A thin metal plate for mice. (not other species). Forceps also work for mice.

old style cage label holder

Car ice scraper

any type of flat/thin instrument (e.g., hemostat, forceps, clamps, etc)

Fingers

Looks like a spatula

plastic scraper

cage lid

Plastic cage scapper

I selected "manual using just fingers", but the survey webpage insists I provide an answer about tools used. So I will comment that some people use metal rods, but this is not the preferred method.

Small ice scraper

Cage divider for a bottle

forceps handles

Handle of a scalpel

plastic cage card, forceps (handle part)

Closed forceps

I don't know the name but it is solid plastic and resembles a scraper that you use to get ice of your car (it is not that thin or sharp, this is just the best comparison).

plastic scraper tool

I use fingers I know others use a cage scraper that's kept specifically for job or label holder

blunt edged metal cage card holder

Plastic scrapper

Syringe

closed forceps

We have metal devices made specifically for this purpose by a workshop in the uni previously. Kind of like a metal cage card holder with a narrow rounded edge and a large rounded edge to hold onto on the other side.

An implement made by our workshop

Stoelting cervical dislocator

closed tweezers

Plastic cage scraper/spatula

Metal plate (may count as rod?)

Purpose made implement

metal cage card holder or custom made metal hand-held plate.

card holders/plastic wallpaper scrapers

plastic spatula, such as might also be used to scrape out dirty cages (the ones used for cervical dislocation are dedicated to only that use)

Metal rod for mice, hand twist method for rats

Scraper

Scraper

ice scraper

metal cage card template. (So not actually a cage card, but something specifically made that resembles a cage card.) Not commonly used though.

Cage card or spatula type tool (unsure of name)

plastic scraper was used previously

Plastic scraper

Cage card

Blunt scrapper

window screen scrapper

Metal plate

scraper

**Q9 Criteria considered important for successful cervical dislocation of mice**

**'Other' criteria responses included:**

Inwards pinch at cervical region from either side of neck

Manipulate region after dislocation to feel that all internal connective tissues have separated.

We use rising concentration of CO2 to euthanise and cerv disc as confirmation, so it is easy to pick up the (already dead) mouse, do a test squeeze of the foot for wincing, then dislocate the neck with your fingers (on a flat surface, squeezing laterally).

Both downwards and forwards simultaneously

Confirmation of cervical dislocation by palpation

A forceful and very quick horizontal push of the tail. The tool used (scissors in my case) only serve to maintain the head so they remain steady along the procedure

We never pull the tail but we do hold it to stop the rodent slipping back.

Clean break confirmation, confirmation of death by feeling heart beat stop

Forceful push of head forward at C1/occipital process. Operators are taught never to hold the tail when killing a mouse

From the scruff hold, push the thumb forward and bring the index finger backwards towards the mouses body to sever vertebrae and connective tissue.

A forceful vertical push towards the surface where the animal is standing

A forceful pinch at the cervical region.

**Q13 Cervical dislocation techniques for laboratory rats**

**'Other' tools responses included:**

Cage card holder

steel scraper

Guillotine

Scalpel blade holder

When it's was up to 500g I used the plastic scrapper. Now it's 150 or sedated I use my fingers as described in the last section

Stoelting cervical dislocator.

tool made specifically for this purpose in house

An implement made by our workshop

Stoelting cervical dislocator

specifically made implement

metal cage card holder or custom made metal hand-held plate.

Card holder

Hand/twist method. No additional implements used

Plastic scraper

Metal rat house

cervical dislocator

Dedicated metal rod for cervical dislocation.

## **<u>Q17 Criteria considered important for successful cervical dislocation of rats</u>**

**‘Other’ criteria responses included:**

Rather than a pull of the tail more the hind legs & tail

Decapitation

forwards and downwards

Placing it on a table with a cloth - you then use your finger to dislocate. Holding the tail is needed but not pulling

In my experience rats and killed with CO2 and then mechanically dislocated using a metal rod as confirmation.

Confidence

**Table S1:** Summary of all the combination of answers obtained from Q9, pertaining to the criteria participants deem important for successful cervical dislocation of mice according to the primary method respondents utilised (manual or mechanical)

| Primary Method | Combination of answers selected for Q9 | n |
|---|---|---|
| Manual | A forceful push downwards at the cervical region of the spine, A forceful push forwards at the cervical region of the spine | 21 |
| Manual | A forceful push forwards at the cervical region of the spine | 13 |
| Manual | A forceful push forwards at the cervical region of the spine, Pulling the tail horizontally | 13 |
| Manual | A forceful push downwards at the cervical region of the spine, A stretch of the region after cervical separation, Pulling the tail horizontally | 12 |
| Manual | A forceful push downwards at the cervical region of the spine, A forceful push forwards at the cervical region of the spine, Pulling the tail horizontally | 11 |
| Manual | A forceful push downwards at the cervical region of the spine | 10 |
| Manual | A forceful push downwards at the cervical region of the spine, Pulling the tail upwards | 9 |
| Manual | A stretch of the region after cervical separation | 9 |
| Mechanical | A forceful push downwards at the cervical region of the spine | 9 |
| Manual | A forceful push downwards at the cervical region of the spine, Pulling the tail horizontally | 8 |
| Manual | A forceful push downwards at the cervical region of the spine, A forceful push forwards at the cervical region of the spine, A stretch of the region after cervical separation | 7 |
| Manual | A forceful push downwards at the cervical region of the spine, A stretch of the region after cervical separation | 7 |
| Manual | A forceful push forwards at the cervical region of the spine, A stretch of the region after cervical separation, Pulling the tail horizontally | 7 |
| Manual | A forceful push forwards at the cervical region of the spine, Pulling the tail upwards | 7 |
| Manual | Other | 7 |
| Manual | Pulling the tail horizontally | 6 |
| Mechanical | A forceful push downwards at the cervical region of the spine, Pulling the tail horizontally | 6 |
| Manual | A forceful push downwards at the cervical region of the spine, A forceful push forwards at the cervical region of the spine, A stretch of the region after cervical separation, Pulling the tail horizontally | 5 |
| Manual | A forceful push downwards at the cervical region of the spine, A forceful push forwards at the cervical region of the spine, Pulling the tail upwards | 5 |
| Manual | A forceful push forwards at the cervical region of the spine, A stretch of the region after cervical separation | 5 |
| Manual | A stretch of the region after cervical separation, Pulling the tail horizontally | 5 |
| Mechanical | A forceful push downwards at the cervical region of the spine, A stretch of the region after cervical separation, Pulling the tail horizontally | 5 |
| Mechanical | Pulling the tail horizontally | 5 |

| | | |
|---|---|---|
| Manual | A forceful push downwards at the cervical region of the spine, A forceful push forwards at the cervical region of the spine, A lateral twist/roll after cervical separation | 4 |
| Manual | A forceful push downwards at the cervical region of the spine, A stretch of the region after cervical separation, Pulling the tail upwards | 4 |
| Manual | A forceful push forwards at the cervical region of the spine, A lateral twist/roll after cervical separation, Pulling the tail horizontally | 4 |
| Mechanical | A forceful push downwards at the cervical region of the spine, A forceful push forwards at the cervical region of the spine, Pulling the tail horizontally | 4 |
| Mechanical | A forceful push downwards at the cervical region of the spine, A stretch of the region after cervical separation, Pulling the tail upwards | 4 |
| Mechanical | A forceful push downwards at the cervical region of the spine, Pulling the tail horizontally, Pulling the tail upwards | 4 |
| Mechanical | A forceful push downwards at the cervical region of the spine, Pulling the tail upwards | 4 |
| Manual | A forceful push downwards at the cervical region of the spine, A lateral twist/roll after cervical separation, A stretch of the region after cervical separation, Pulling the tail horizontally | 3 |
| Manual | A forceful push forwards at the cervical region of the spine, A lateral twist/roll after cervical separation | 3 |
| Manual | A forceful push forwards at the cervical region of the spine, A lateral twist/roll after cervical separation, Pulling the tail upwards | 3 |
| Manual | A lateral twist/roll after cervical separation | 3 |
| Mechanical | A forceful push downwards at the cervical region of the spine, A forceful push forwards at the cervical region of the spine | 3 |
| Mechanical | A forceful push downwards at the cervical region of the spine, A forceful push forwards at the cervical region of the spine, Pulling the tail upwards | 3 |
| Mechanical | A forceful push downwards at the cervical region of the spine, A stretch of the region after cervical separation | 3 |
| Mechanical | A forceful push downwards at the cervical region of the spine, A stretch of the region after cervical separation, Pulling the tail horizontally, Pulling the tail upwards | 3 |
| Mechanical | A forceful push forwards at the cervical region of the spine, Pulling the tail horizontally | 3 |
| Manual | A forceful push downwards at the cervical region of the spine, A forceful push forwards at the cervical region of the spine, A lateral twist/roll after cervical separation, A stretch of the region after cervical separation | 2 |
| Manual | A forceful push downwards at the cervical region of the spine, A forceful push forwards at the cervical region of the spine, A lateral twist/roll after cervical separation, Pulling the tail horizontally | 2 |
| Manual | A forceful push downwards at the cervical region of the spine, A forceful push forwards at the cervical region of the spine, A stretch of the region after cervical separation, Pulling the tail upwards | 2 |
| Manual | A forceful push downwards at the cervical region of the spine, A lateral twist/roll after cervical separation | 2 |
| Manual | A forceful push downwards at the cervical region of the spine, A lateral twist/roll after cervical separation, A stretch of the region after cervical separation | 2 |

| | | |
|---|---|---|
| Manual | A forceful push downwards at the cervical region of the spine, A lateral twist/roll after cervical separation, Pulling the tail horizontally | 2 |
| Manual | A forceful push forwards at the cervical region of the spine, A lateral twist/roll after cervical separation, A stretch of the region after cervical separation, Pulling the tail upwards | 2 |
| Manual | A forceful push forwards at the cervical region of the spine, A stretch of the region after cervical separation, Pulling the tail upwards | 2 |
| Mechanical | A forceful push downwards at the cervical region of the spine, A forceful push forwards at the cervical region of the spine, A stretch of the region after cervical separation | 2 |
| Mechanical | A forceful push downwards at the cervical region of the spine, A forceful push forwards at the cervical region of the spine, A stretch of the region after cervical separation, Pulling the tail horizontally | 2 |
| Mechanical | A forceful push downwards at the cervical region of the spine, A forceful push forwards at the cervical region of the spine, Pulling the tail horizontally, Pulling the tail upwards | 2 |
| Mechanical | A forceful push forwards at the cervical region of the spine | 2 |
| Mechanical | A forceful push forwards at the cervical region of the spine, A stretch of the region after cervical separation, Pulling the tail horizontally | 2 |
| Mechanical | A forceful push forwards at the cervical region of the spine, A stretch of the region after cervical separation, Pulling the tail upwards | 2 |
| Mechanical | A stretch of the region after cervical separation | 2 |
| Mechanical | A stretch of the region after cervical separation, Pulling the tail upwards | 2 |
| Manual | A forceful push downwards at the cervical region of the spine, A forceful push forwards at the cervical region of the spine, A lateral twist/roll after cervical separation, A stretch of the region after cervical separation, Pulling the tail horizontally | 1 |
| Manual | A forceful push downwards at the cervical region of the spine, A forceful push forwards at the cervical region of the spine, A lateral twist/roll after cervical separation, Pulling the tail upwards | 1 |
| Manual | A forceful push downwards at the cervical region of the spine, A forceful push forwards at the cervical region of the spine, A stretch of the region after cervical separation, Pulling the tail horizontally, Pulling the tail upwards | 1 |
| Manual | A forceful push downwards at the cervical region of the spine, A lateral twist/roll after cervical separation, A stretch of the region after cervical separation, Pulling the tail upwards | 1 |
| Manual | A forceful push downwards at the cervical region of the spine, A lateral twist/roll after cervical separation, Pulling the tail upwards | 1 |
| Manual | A forceful push downwards at the cervical region of the spine, A stretch of the region after cervical separation, Pulling the tail horizontally, Pulling the tail upwards | 1 |
| Manual | A forceful push downwards at the cervical region of the spine, Other | 1 |
| Manual | A forceful push downwards at the cervical region of the spine, Other, Pulling the tail upwards | 1 |
| Manual | A forceful push downwards at the cervical region of the spine, Pulling the tail horizontally, Pulling the tail upwards | 1 |
| Manual | A forceful push forwards at the cervical region of the spine, A lateral twist/roll after cervical separation, A stretch of the region after cervical separation | 1 |
| Manual | A forceful push forwards at the cervical region of the spine, A lateral twist/roll after cervical separation, A stretch of the region after | 1 |

| | | |
|---|---|---|
| | cervical separation, Pulling the tail horizontally, Pulling the tail upwards | |
| Manual | A forceful push forwards at the cervical region of the spine, A stretch of the region after cervical separation, Pulling the tail horizontally, Pulling the tail upwards | 1 |
| Manual | A lateral twist/roll after cervical separation, A stretch of the region after cervical separation, Pulling the tail horizontally | 1 |
| Manual | A lateral twist/roll after cervical separation, Pulling the tail upwards | 1 |
| Manual | A stretch of the region after cervical separation, Pulling the tail upwards | 1 |
| Manual | Other, Pulling the tail horizontally | 1 |
| Manual | Pulling the tail upwards | 1 |
| Mechanical | A forceful push downwards at the cervical region of the spine, A forceful push forwards at the cervical region of the spine, A lateral twist/roll after cervical separation, A stretch of the region after cervical separation, Pulling the tail horizontally | 1 |
| Mechanical | A forceful push forwards at the cervical region of the spine, A lateral twist/roll after cervical separation, A stretch of the region after cervical separation | 1 |
| Mechanical | A forceful push forwards at the cervical region of the spine, A stretch of the region after cervical separation | 1 |
| Mechanical | A forceful push forwards at the cervical region of the spine, Pulling the tail horizontally, Pulling the tail upwards | 1 |
| Mechanical | A forceful push forwards at the cervical region of the spine, Pulling the tail upwards | 1 |
| Mechanical | A lateral twist/roll after cervical separation | 1 |
| Mechanical | A stretch of the region after cervical separation, Pulling the tail horizontally | 1 |
| Mechanical | Other | 1 |
| Mechanical | Other, Pulling the tail horizontally | 1 |
| Mechanical | Pulling the tail horizontally, Pulling the tail upwards | 1 |
